# Stem cell proliferation during *in vitro *development of the model cestode *Mesocestoides corti *from larva to adult worm

**DOI:** 10.1186/1742-9994-7-22

**Published:** 2010-07-13

**Authors:** Uriel Koziol, María F Domínguez, Mónica Marín, Alejandra Kun, Estela Castillo

**Affiliations:** 1 Sección Bioquímica y Biología Molecular, Facultad de Ciencias, Universidad de la República, Iguá 4225, CP 11400, Montevideo, Uruguay; 2 Departamento de Proteínas y Ácidos Nucleicos, Instituto de Investigaciones Biológicas Clemente Estable, Avenida Italia 3318, CP11600, Montevideo, Uruguay

## Abstract

**Background:**

In free-living flatworms somatic differentiated cells do not divide, and a separate population of stem cells (called neoblasts) is responsible for cell proliferation and renewal. In cestodes, there is evidence that similar mechanisms of cell renewal exist.

**Results:**

In this work, we have characterized proliferative cells during the development of the model cestode *Mesocestoides corti *from larva (tetrathyridium) to young segmented worm. This was done by two complementary strategies with congruent results: characterizing cells in S phase and their progeny by incorporation of 5-bromo-2'-deoxyuridine, and characterizing cells in M phase by arresting mitotic cells with colchicine and studying their morphology and distribution. Proliferative cells are localized only in the inner parenchyma, particularly in close proximity to the inner muscle layer, but not in the cortical parenchyma nor in the sub-tegumental tissue. After proliferation some of these cells migrate to the outer regions were they differentiate. In the larvae, proliferative cells are more abundant in the anterior regions (scolex and neck), and their number diminishes in an antero-posterior way. During the development of adult segments periodic accumulation of proliferative cells are observed, including a central mass of cells that constitutes the genital primordium, which grows at least in part due to *in situ *proliferation. In later segments, the inner cells of genital primordia cease to proliferate and adopt a compact distribution, and proliferative cells are also found in the testes primordia.

**Conclusions:**

Proliferative cells have a characteristic localization and morphology throughout development from larva to adult of *Mesocestoides corti*, which is similar, and probably evolutionary conserved, to that described in other model cestodes. The characteristics of proliferative cells suggest that these consist of undifferentiated stem cells.

## Background

In free-living platyhelminthes, the best studied model being planarians, somatic differentiated cells do not divide and a separate cellular population of stem cells, called neoblasts, are responsible for cell proliferation and renewal during growth, regeneration and maintenance [1-4]. Recently, the study of planarian cell proliferation has been revolutionized by new cellular and molecular biology approaches which have allowed considerable insight into the mechanisms of neoblast maintenance and differentiation, and into the existence of different sub-populations of neoblasts and their progeny [3,5-7]. In the parasitic clade Neodermata, which includes the well known classes Cestoda, Monogenea and Trematoda, there is evidence that similar mechanisms of cell renewal exist [2]. This has been studied mostly in cestodes, in which the functional equivalents of neoblasts are usually referred to as germinative cells.

*Diphyllobothrium dendriticum *(Pseudophyllidea) is probably the cestode species in which the characterization of germinative cells has been most thorough, as well as their differentiation into different cell types during histogenesis [8-12]. Germinative cells in the plerocercoid larva and adult of *D. dendriticum *and other *Diphyllobothrium *species are absent in the outer regions of the cortical parenchyma and sub-tegumental tissue, and are localized mainly in the inner regions of cortical parenchyma and in the medullary parenchyma. They are especially abundant in close proximity to the inner muscle layer. These cells migrate from the inner parenchyma to the outer parenchyma and sub-tegumental tissue for cell renewal and tissue growth. During strobilization, they accumulate in the genital primordium in each segment. Localization and characterization of proliferative cells has also been studied to different degrees in other cestode models, with generally similar results, although some differences were observed in larval stages of *Taenia *and *Hymenolepis*, were proliferative cells are not restricted to the medullary parenchyma [13-22]. The morphology of germinative cells is very characteristic, since they are undifferentiated cells with round shape, a large nucleus with little heterochromatin and a very prominent nucleolus, and a very basophilic cytoplasm due to the abundance of RNA. Ultrastructural studies demonstrate the abundance of free ribosomes and the absence or paucity of endoplasmic reticulum and Golgi apparatus [9,18].

*Mesocestoides corti *is a model for studying cestode biology and development. It is particularly interesting because of its intermediate larval stage (tetrathyridium), composed of a scolex and an unsegmented body, which is uniquely able to proliferate asexually by longitudinal fission in the peritoneum and organs of mice and several other intermediate hosts. This allows the maintenance of large and constant populations of worms through repeated serial intraperitoneal passage in mice [23]. *In vitro *culture of tetrathyridia allows studying either asexual reproduction or segmentation (formation of successive segments in the body) and strobilar development (formation of serially arranged genital organs, one in each segment), depending on the culture conditions used [24-27]. Strobilar development is very similar *in vitro *and *in vivo*, making it an ideal model for studying this process [26] except that viable eggs have only been sporadically documented.

The first studies on cellular proliferation in *M. corti *were done in tetrathyridia by Hess [28-30]. Initially, large basophilic cells were identified by histological methods (methyl green/pyronin) on whole-mount material as possible germinative cells. In later electron microscopy studies, Hess proposed that at least two different proliferative cell populations exist in tetrathyridia. One is a population of germinative cells in the parenchyma (which would be similar to the localization of germinative cells in other cestodes), for which he found evidence of differentiation into parenchymal muscle cells, but not into sub-tegumental muscle cells or tegumental cells (also known as perinuclear cell bodies of the tegumental syncytium). The other is a reticulated syncytium of cells in connection with the tegument (and therefore part of the tegumental syncytium), located between the suckers, where longitudinal fission begins, and termed by Hess as the 'apical massif'. In this tissue Hess observed mitotic figures and incorporation of tritiated thymidine. Proliferating tegumental cells were not observed in other regions, and have not been described in any other cestode. He proposed that the apical massif is a major source of differentiating cells during asexual reproduction. Furthermore, he proposed, based on ultrastructural similarities, that cells from the apical massif detach from it and migrate to the tegumental region where they are incorporated for growth of the tegumental syncytium during normal growth.

Smith and McKerr [31], in a mainly methodological work, demonstrated the utility of the thymidine analog, 5-bromo-2'-deoxyuridine (BrdU), for labeling cells in S phase in tetrathyridia. Using a 24 hours pulse, they found labeled cells throughout the parenchyma, not being especially abundant in the region between the suckers. They also found labeled cells in the sub-tegumental region. However, the length of the pulse could allow for cell migration in this case.

Finally, Espinoza and collaborators [32,33] performed autoradiographic analysis of cells incorporating tritiated thymidine in tetrathyridia and during segmentation. Although the purpose of the autoradiographic analysis was mainly for quantification of cell proliferation, demonstrating important increases during early and late segmentation, they also described an abundance of labeled cells close to the region between internal and external parenchyma, interpreting this as incorporation of proliferative cells into the nerve cords. However, this was also done with 24 hours pulses exclusively, and only longitudinal sections were shown as a basis for this interpretation. The localization of proliferative cells during the formation and development of genital primordia was not described.

In this work, we have thoroughly characterized the localization and abundance of proliferative cells during the *in vitro *development of *M. corti *from tetrathyridium to young segmented worm. This was done by two complementary strategies: characterizing cells in S phase and their progeny by pulse and pulse-chase experiments with BrdU (including short pulses of only 4 hours to prevent cell migration), and characterizing cells in M phase by arresting mitotic cells with colchicine and studying their morphology and distribution.

## Results

### Description of early proglottid formation in *M. corti*

Strobilar development of *M. corti *has been described both *in vivo *and *in vitro *[25,26,34], but the first stages of development of the genital primordia have not been characterized in detail. We therefore performed studies on whole-mount specimens stained with TO-PRO-3, a nuclear stain, focusing our attention in the early development of proglottids and genital primordia.

**Figure 1:**
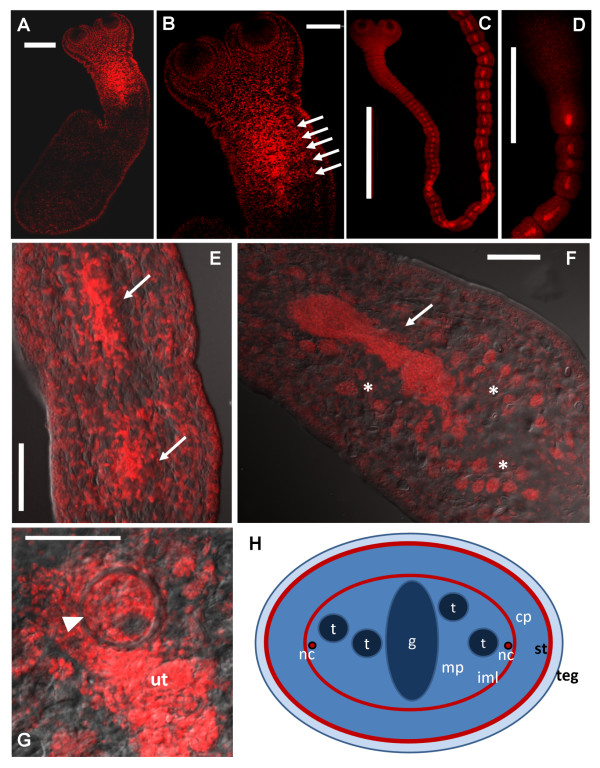
**Morphology of segments and genital primordia of *M. corti *during early segmentation. **All specimens were stained with TO-PRO-3. A. Tetrathyridium beginning segmentation. B. Close-up of early cell accumulations in segment primordia (arrows) with central genital primordia. C. Developed adult worm. D. Posterior region of the same adult worm as in C; anterior is to the bottom; notice the lack of segmentation of the posterior most region. E. Early segments of adult worm, showing early genital primordia (arrows). F. Later segment of adult worm showing late genital primordium (arrow) and testis primordia (asterisks). G. Later segment of adult worm showing cirrus pouch (arrowhead) and developing uterus (*ut*). H. Schematic drawing of a transverse section of a segment with late genital primordium. *cp*, cortical parenchyma; *g*, genital primordium; *iml*, inner muscle layer; *mp*, medullary parenchyma; *nc*, main nerve cords; *st*, sub-tegumental region with sub-tegumental muscle layer; *t*, testes primordia; *teg*, tegument (which consists of a syncytium that covers the body in a continuous sheet connected to perikarya (tegumental cells) that lie below the sub-tegumental muscle layer [43]). Bars represent 200 μm in A, 100 μm in B and F, 1000 μm in C and D, and 50 μm in E and G.

After induction with sodium taurocholate (ST), tetrathyridia elongate, and the neck region, which becomes narrower than the scolex, shows a great density of cells. In a first stage of development, several segment primordia (approximately 6), with their respective genital primordia, can be observed behind the neck (Figure 1A, B). These genital primordia consist of small and loose accumulations of cells in the center of each segment. The posterior-most region of tetrathyridia, where the excretory pore or pores are located (since one pore is formed after each asexual reproduction cycle [35]), shows a much lower cell density, a higher abundance of calcareous corpuscles, and does not participate in the segmentation process. In more developed specimens, an antero-posterior gradient of development is observed, since new segments are being formed continuously in the neck region (Figure 1C), but even in these specimens the posterior-most region does not segment nor elongate (Figure 1D). Initially, in proglottids next to the neck region, the genital primordium is small and cells are loosely packed (Figure 1E). In posterior segments, it grows considerably, while at the same time the primordia of the testes appear to its sides. The genital primordium continues to grow, and becomes a very compact and elongated mass of cells, which divides in an anterior and a posterior region (Figure 1F), presumably the male and female genital rudiments respectively. In some but not all cases, a developed cirrus pouch and developing uterus can already be observed around this stage (Figure 1G).

### Detection of BrdU incorporation as a marker for cells in S phase

The distribution of cells in S phase was determined by the detection of BrdU incorporation in whole-mount specimens and sections of uninduced tetrathyirida, tetrathyridia after ST induction, and in segmented worms. Two different pulse lengths were used: a 24 hour pulse and a 4 hour pulse. The 24 hour pulse should label all actively proliferating cells (taking into account the length of the cell cycle estimated in other cestode species, 8.5 hours in 4 day old *Hymenolepis diminuta*, and 19 hours in *Diphyllobothrium dendriticum *plerocercoids; [18,36]), and the results can be directly compared to previous studies in tetrathyridia [31]. However, it could be long enough to allow some labeled cells to migrate and initiate differentiation. The 4 hour pulse, on the other hand, is shorter or similar in length to the S phase in other cestodes [18,36], and would only label a fraction of actively proliferating cells, being short enough to prevent significant migration.

### BrdU positive cells after a 24 hour BrdU pulse

**Figure 2:**
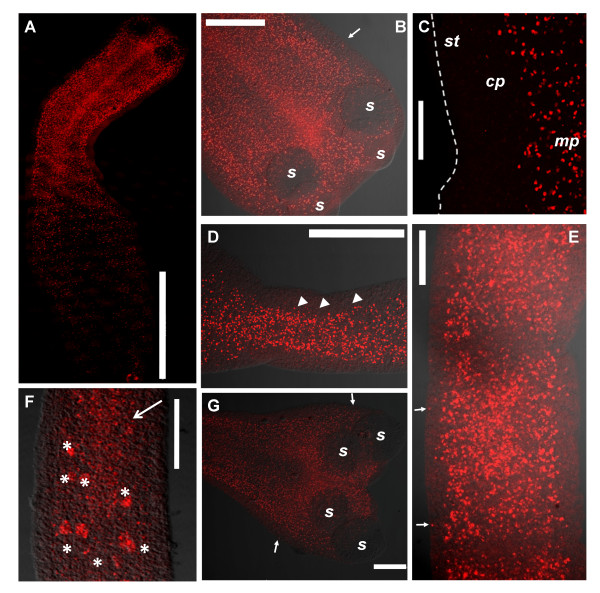
**Whole-mount BrdU detection after a 24 hour pulse of ST induced specimens. **A. Full view of a tetrathyridium. B. Close-up of the scolex of the specimen shown in 2A. C. Close-up of the border of the body wall of a tetrathyridium. The broken line indicates the limit of the body wall. D. Initiation of segmentation; arrowheads indicate periodic accumulations of BrdU+ cells. Anterior is to the right. E. Early segments of adult worm. F. Later segment of adult worm, with genital primordium (large arrow) and testis primordia (asterisks). G. Scolex of adult worm. Small arrows in all figures indicate rare BrdU+ cells in the cortical parenchyma. *cp*, cortical parenchyma; *mp*, medullary parenchyma; *s*, sucker; *st*, sub-tegument. Note that the tegument was previously removed. Bars represent 1000 μm in A, 200 μm in B, D and G, 50 μm in C and E, and 100 μm in F.

BrdU positive (BrdU+) cells show a very similar distribution in tetrathyridia both before and after induction with ST (Figure 2A-C, [see Additional File 1]). They show an accumulation in the scolex and the entire anterior-most region, particularly next to and behind the suckers. The region between the suckers (where the apical massif is located) shows a high number of BrdU+ cells, but not higher than in other regions next to the suckers. BrdU+ cells can also be detected within the suckers.

There is a clear antero-posterior gradient in the distribution of BrdU+ cells; there are few BrdU+ cells in the posterior-most region, which does not participate in segmentation. BrdU+ cells after a 24 hour pulse are localized throughout the medullary parenchyma, particularly in its periphery, next to the inner muscle layer, as determined by analysis of several focal planes. No evidence was observed of preferential accumulation of BrdU+ cells in the region of the main nerve cords. Very few BrdU+ cells can be observed in the cortical parenchyma and sub-tegumental region (outside of the inner muscle layer), both in the scolex as in the body. These few cells probably acquired the label while in the medullary region, and then migrated to the cortical and sub-tegumental regions (see below).

In very early segmenting specimens, periodic accumulations of BrdU+ cells can be observed, even in the absence of external signs of segmentation (Figure 2D). In segmented specimens, BrdU+ cells are also found in the medullary region, with very few BrdU+ cells in the cortical and sub-tegumental regions (Figure 2E-G). A large number of BrdU+ cells are found in the scolex and in the neck. In early proglottids, BrdU+ cells are segmentally distributed and accumulated in the early genital primordium. In more developed proglottids, strongly labeled BrdU+ cells are found in the late genital primordium and in the testes primordia.

**Figure 3:**
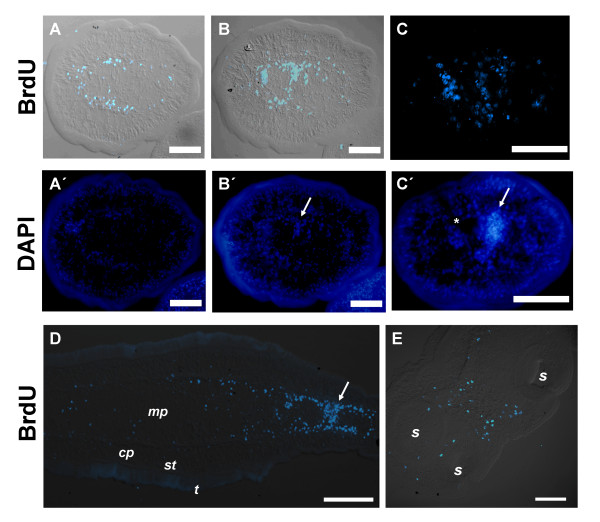
**BrdU detection in sections of segmented worms after a 24 hour pulse. **A, A', B, B' and C, C'. Transverse sections of early (A, B) and later (C) segments stained with anti-BrdU and DAPI. Arrow indicates genital primordia, and asterisks testis primordia. D. Sagittal section of early segmenting worm, showing last genital primordium (arrow) and the posterior non segmenting region. Anterior is to the right. E. Transverse section of the scolex. *cp*, cortical parenchyma; *mp*, medullary parenchyma; *s*, sucker; *st*, sub-tegument; *t*, tegument. Bars represent 50 μm except in D, 100 μm.

BrdU detection in sections was performed in order to study in greater detail the localization of proliferating cells in segmenting worms (Figure 3). In transverse sections, it is readily apparent that BrdU+ cells are mainly distributed in the medullary parenchyma, particularly in positions next to the inner muscle layer, thus forming a ring shape in cross-sections (Figure 3A-C). No accumulations were apparent in the region of the main nerve cords [37-39]. Some BrdU+ cells are found in the base of the cortical parenchyma, in direct contact with the inner muscle layer. In the posterior-most region, although the number of BrdU+ cells is smaller, their distribution is very similar (data not shown). Similar results were observed in serial sagittal sections (Figure 3D); the observation of several consecutive sections was important to confirm the presence of BrdU+ cells throughout the external region of the medullary parenchyma. In the scolex, BrdU+ cells are also found mainly in the outer regions of the medullary parenchyma, close to the inner muscle layer and to muscle fibers attaching suckers to each other (originally described by Terenina *et al*. [37]; Figure 3E). BrdU+ cells can also be observed within the suckers. Only two BrdU+ cells were found in the cortical parenchyma and sub-tegumental regions in over 40 sections observed, confirming the results obtained with whole-mount material.

The early genital primordium is formed by a loose accumulation of BrdU+ cells that traverses dorso-ventrally the medullary parenchyma, and which is continuous with thickenings of the ring of BrdU+ cells in the periphery of the medullary parenchyma. In later proglottids, the size of the primordia increases, consisting of an internal compact region and an external loose region (Figure 3C'). Only cells in the external region are labeled by BrdU. Strong BrdU labeling can also be detected in some but not all primordia of testes. Within an individual testis primordium, labeling is similar among all cells (data not shown). These results suggest synchronic proliferation of the cells of the testes primordia.

### BrdU positive cells after a 4 hour BrdU pulse

**Figure 4:**
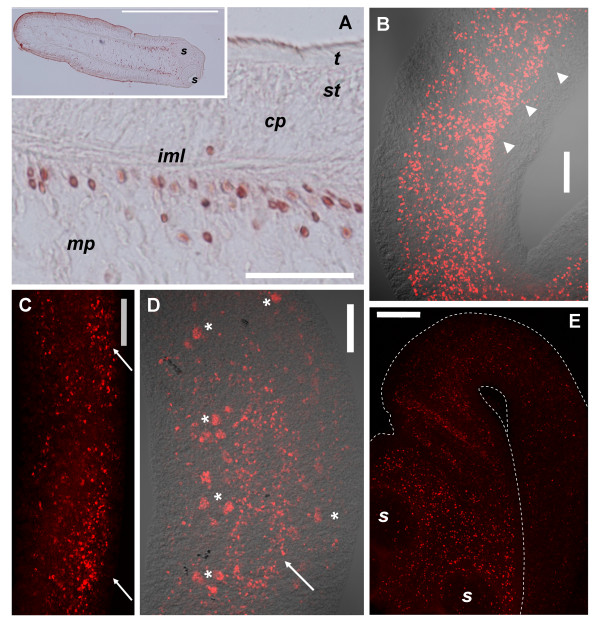
**BrdU detection after a 4 hour pulse. **A. Sagittal section of a tetrathyridium. The inset shows a general view of the same specimen. B. Whole-mount of early segmenting specimen showing periodic accumulations of BrdU+ cells (arrowheads). C. Early segments of whole-mount adult worm (lateral view) with early genital primordia (arrows). D. Later segment of whole-mount adult worm, with late genital primordium (arrow) and testis primordia (asterisks). E. Scolex and neck of adult worm; the broken line indicates the limit of the body wall. *cp*, cortical parenchyma; *iml*, inner muscle layer; *mp*, medullary parenchyma; *s*, sucker; *st*, sub-tegument; *t*, tegument. Note that the tegument was removed in whole-mount specimens. Bars represent 20 μm in A (200 μm in the inset), 50 μm in B, C and D, and 100 μm in E.

In experiments in which a 4 hour BrdU pulse was performed, no BrdU+ cells were found in the cortical or sub-tegumental regions in any of the developmental stages, confirming the absence of proliferation in these regions (Figure 4). This strongly indicates that the BrdU+ cells found in these regions after 24 hour long BrdU pulses originated in the medullary parenchyma and later migrated to the outer regions.

Periodic accumulations of BrdU+ cells were observed in segmenting worms before any external sign of segmentation was apparent, indicating that this accumulation occurs at least in part due to *in situ *proliferation (Figure 4B). In more developed worms, abundant BrdU+ cells occur in the scolex and neck, followed by smaller numbers in the region immediately posterior to the neck, just before segments are evident (Figure 4E). This is expected, since the neck region is known to be the proliferating region generating the proglottids in other cestodes [2]. Large amounts of BrdU+ cells were also found in early and late genital primordia, and in the primordia of testes, confirming the existence of abundant *in situ *proliferation in these regions (Figure 4C, D). In late genital primordia, BrdU+ cells are found mainly in their periphery, as observed in sections after 24 hour long BrdU pulses (see above). This indicates that growth occurs mainly by proliferation of cells in the periphery of the primordium, although a contribution of immigrating parenchymal cells cannot be discarded.

### BrdU positive cells after pulse-chase experiments

**Figure 5:**
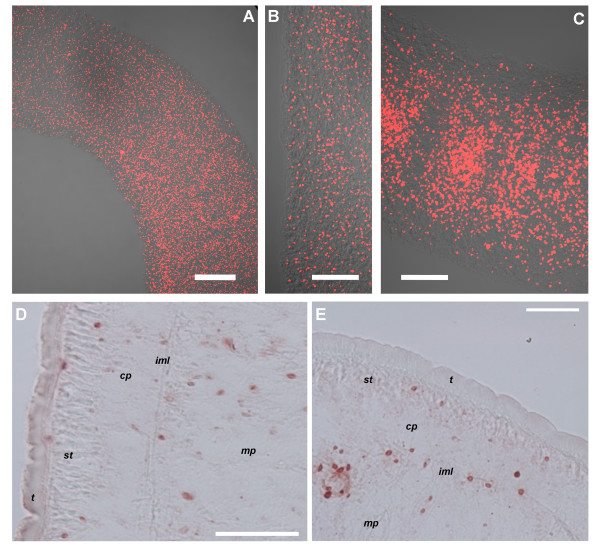
**BrdU detection in pulse and chase and in continuous labeling experiments. **A, B. Whole-mount detection of BrdU in a tetrathyridium after a 24 hour pulse followed by a 48 hour chase. C. Whole-mount detection of BrdU in early segments of an adult worm after a 24 hour pulse followed by a 48 hour chase. D. Sagittal section of tetrathyridium after a 4 hour pulse followed by a 68 hour chase. E. Section of tetrathyridium with an amputated scolex after 72 hours of continuous labeling with BrdU. *cp*, cortical parenchyma; *iml*, inner muscle layer; *mp*, medullary parenchyma; *st*, sub-tegument; *t*, tegument. Note that the tegument was removed in whole-mount specimens. Bars represent 100 μm in A, 50 μm in B and C, and 20 μm in D and E.

The absence of proliferating cells in the cortical parenchyma and sub-tegumental tissues strongly suggested that cell renewal and growth in these regions occurs by immigration of proliferating cells from the medullary parenchyma, similar to what has been described in other cestodes [8,19]. Furthermore, Hess [30] reported the absence of mitoses in the sub-tegumental region. In order to confirm this hypothesis, we performed labeling experiments with either a 4 or 24 hour BrdU pulse, followed by a two to three day chase in BrdU-free media (Figure 5). Indeed, in tetrathyiridia after the chase, BrdU+ cells are distributed almost homogeneously, including the cortical parenchyma and sub-tegumental tissues, confirming this hypothesis, and indicating that between 24 and 72 hours are required for migration of most cells to these regions (Figure 5A, B, E). In segmented specimens, many BrdU+ cells are found after the 48 hours chase in the cortical parenchyma and sub-tegumental tissues (Figure 5C), especially in the scolex and neck (data not shown). However, the number is much lower than in tetrathyridia (compare figures 5A and 5C), and most BrdU+ cells in the proglottids are localized in the genital and testes primordia. This suggests that during segmentation, cell renewal in the external regions is diminished, and that the destiny of most proliferative cells is the development of reproductive structures.

### BrdU positive cells after continuous labeling in tetrathyridia with an experimentally amputated scolex

Hess [30], based on ultrastructural studies, suggested that cells integrating into the tegumental syncytium were originated by proliferation in the apical massif, from where they detach and migrate to the sub-tegumental region for fusion with the tegument. This mode of sub-tegumental replacement was proposed to occur during asexual reproduction and during normal growth. The apical massif was also proposed as the source of new sub-tegumentary muscle cells.

Furthermore, it was indicated that germinative cells did not contribute to either tegumental cells or sub-tegumental muscle cells. However, our results with short BrdU pulses and pulse chase experiments suggest that cells integrating into the sub-tegumental region could originate throughout the body, as suggested by Smith and McKerr [31]. We tested this hypothesis by experimentally amputating the scolex region in tetrathyridia (therefore removing the apical massif). These fragments do not regenerate [40], but remain viable, similarly to natural acephalic fragments [24,40]. After two days of culture, these fragments were subject to continuous labeling with BrdU during three days. Under these conditions, although labeled cells were few (which is not surprising, since proliferation in the posterior region is always scarce), labeled nuclei were observed in the sub-tegumental region, where only cells of the tegumental syncytium and sub-tegumentary muscle cells are located [30] (Figure 5E). Therefore, cells proliferating in the medullary parenchyma can be the source of cells incorporating into the sub-tegumental region.

### Identification of cells in M phase

**Figure 6:**
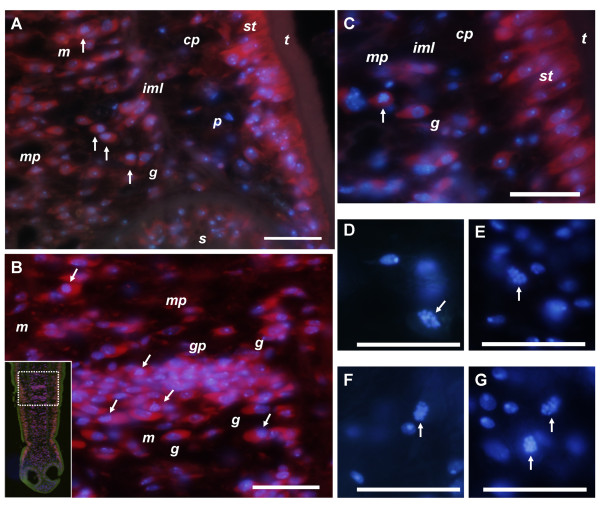
**Histological detection of mitoses in colchicine treated specimens stained with ethidium bromide (red) and/or DAPI (blue). **A. Sagittal section of the region directly behind the suckers. Anterior is to the bottom. B. Sagittal section of early genital primordium. The inset shows a general view of the same specimen. C. Close-up of germinative cells in the body of a tetrathyridium. D, E, F, G. Close-up of mitotic figures stained only with DAPI. Arrows in all figures indicate mitotic cells. *cp*, cortical parenchyma; *g*, germinative cells; *gp*, genital primordium; *iml*, inner muscle layer; *m*, dorso-ventral muscle cell; *mp*, medullary parenchyma; *p*, parenchymal cell; *st*, sub-tegument; *t*, tegument. Bars represent 10 μm.

As a parallel approach, we determined the localization of cells in mitosis in tetrathyridia and in tetrathyridia initiating the process of segmentation. To this end, specimens were cultured for 6 hours in medium with 0.05% colchicine, which arrests mitotic cells in metaphase, allowing the mitotic figures to accumulate. This method has been very useful for the identification and localization of mitotic figures in other cestodes [9,18,20]. Although conventional haematoxylin staining did not allow us to discern mitotic figures (not shown), probably because of the very small size of the chromosomes and the basophilic character of germinative cells in cestodes [9], these were readily identifiable in sections stained with 4',6-diamidino-2-phenylindole (DAPI) dilactate (Figure 6). Sections were co-stained with ethidium bromide, in order to identify cells with RNA rich cytoplasm. This allowed us to observe cell morphology and is analogous to the basophilic staining of the cytoplasm in RNA rich cells under conventional histological techniques, such as methyl green/pyronin [9,28]. Identification of cell types was based on the criteria and descriptions of Douglas [16], Gustafsson [12], Loehr and Mead [15], and Hess [28].

Cells similar to the germinative cells described in other cestodes were observed (containing a large nucleus with finely granular chromatin, large prominent nucleolus, rounded undifferentiated shape and cytoplasm strongly stained with ethidium bromide). These were located in the medullary parenchyma and accumulated in genital primordia (Figure 6, [see Additional File 2]). With the exception of mitotic cells found in the suckers (see below), cells found in mitosis were of similar size to these putative germinative cells, and were intensely stained with ethidium bromide. This suggests that almost all proliferating cells are of the germinative cell type.

**Figure 7:**
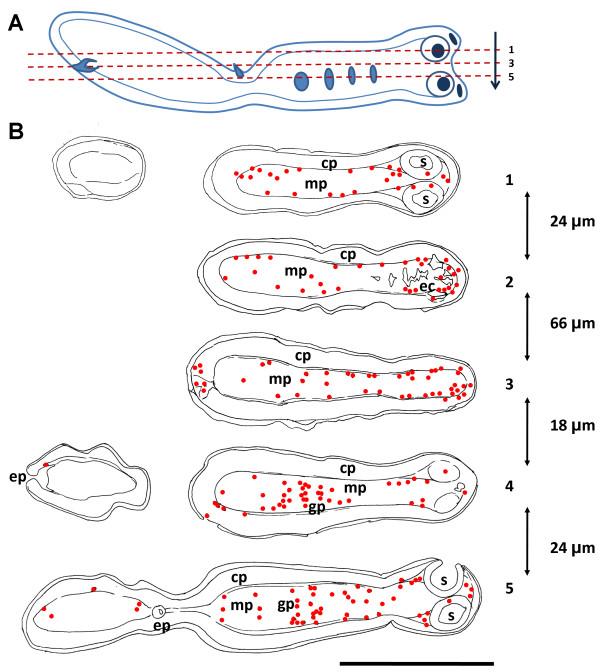
**Position of mitotic cells in serial sagittal sections of *M. corti *at the beginning of segmentation. **A. Schematic drawing of one analyzed specimen, indicating the orientation and direction of the successive planes of section. Numbers of planes correspond to illustrations in part B. B. Scale drawings of some of the analyzed sections from that specimen, indicating the position of mitotic figures (red dots). The approximate distance between sections is indicated to the right. In sections 2 and 3, the posterior-most region (which, due to the curvature of the specimen, is not continuous in these sections with the anterior-most region) has no mitotic figures and is not illustrated. The only mitotic cell found in the cortical region (in over 600 identified mitoses) is illustrated in section 4. Notice the proximity of this cell to an accessory excretory pore. *cp*, cortical parenchyma; *ec*, excretory ducts; *ep*, excretory pore; *gp*, genital primordium; *mp*, medullary parenchyma. The bar represents approximately 200 μm.

**Figure 8:**
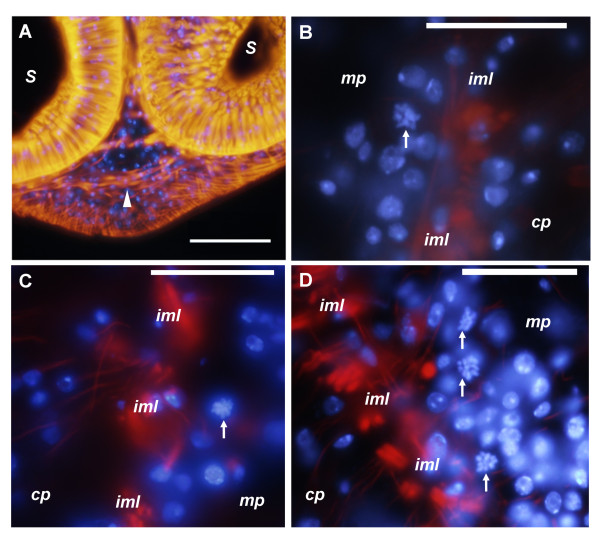
**Close proximity of mitotic figures to the inner muscle layer. **Specimens were stained with DAPI (blue) and phalloidin (orange) A Sagittal section of scolex of tetrathyridium showing the dorso-ventral muscles anterior to the suckers (arrowhead). B, C, D. Close-ups of mitotic cells next to the inner muscle layer. *cp*, cortical parenchyma; *iml*, inner muscle layer; *mp*, medullary parenchyma; *s*, sucker. Arrows in all figures indicate mitotic cells. Bars represent 20 μm in A, and 10 μm in B, C and D.

The localization of cells in M phase is very similar to the localization of cells in S phase as determined by BrdU incorporation (Figure 7). Mitotic figures are more abundant in the scolex and anterior regions of the body, while very few exist in the posterior region which does not segment. In the body, mitotic figures were found in the medullary parenchyma, especially in close proximity to the inner muscular layer, although many also occur in the inner regions. Mitotic figures also occur in the innermost region of the cortical parenchyma, in direct contact with the inner muscular layer. The close proximity of mitotic figures and the inner muscle layer was confirmed by double labeling with DAPI and Alexa-546 conjugated phalloidin which strongly stains the inner and sub-tegumental muscle layers (Figure 8; [37]). Only one mitotic figure was found close to the sub-tegumental region, as part of an accumulation of cells associated to an accessory excretory pore.

In the scolex, mitotic figures are most abundant between and directly behind the suckers, always in close proximity to the inner muscular layer. Mitotic cells also occur within the suckers, confirming the existence of proliferating cells within these organs. However, these cells show a differentiated morphology in many cases [see Additional File 3], since the cytoplasm usually shows extensions and is less stained with ethidium bromide. In the region anterior to the suckers, and between the pairs of suckers, where the apical massif was described [30], many mitoses exist, but most of them are next to the inner muscle layer that continues from the suckers as dorso-ventral muscles, previously described by Terenina *et al. *[37] (Figure 7, Figure 8A, [see Additional File 4]). It is possible that some of these mitotic cells are part of the apical massif as described by Hess [30], but most of them are distant from the sub-tegumental region.

In early genital primordia, germinative cells accumulate and mitotic figures are common (Figure 6C, Figure 7), even in the absence of colchicine incubation (not shown), confirming the existence of *in situ *proliferation. In larger genital primordia, cells have a more compact distribution, more differentiated cells are observed, and in some cases mitoses are not apparent in their interior (data not shown).

## Discussion

### Localization of proliferative cells in *M. corti*

Proliferating cells in *M. corti *are localized in the medullary parenchyma, mainly in the periphery, close to the inner muscle layer. Proliferative cells in the scolex show a similar distribution, close to the muscle layers and in the muscular suckers. Proliferating cells are absent from the cortical parenchyma and sub-tegumental regions. Cell renewal and growth in these regions is achieved by immigration of cells from the medullary parenchyma. Morphologically, cells with characteristics similar to the germinative cells of other cestodes, and previously described as "basophilic cells" by Hess [28], are present in the medullary parenchyma and are the only cells observed in mitosis, with the exception of the suckers.

In tetrathyridia, the abundance of proliferating cells is greater in the anterior region (scolex and neck), while in the posterior region, which is rich in calcareous corpuscles and does not participate of segmentation, proliferating cells are scarce. This is consistent with *in vivo *studies, where this region has been shown to be shed by tetrathyridia in the definitive host, and sometimes in the intermediate host, not participating in either segmentation nor in asexual reproduction by longitudinal fission [35,41]. It is also consistent with the absence of asexual reproduction by budding from this region, as proposed by Hart [38] and Novak [35], unlike the original interpretation by Specht and Voge [23]. In segmented specimens, proliferating cells are more abundant in the scolex and neck than in early proglottids. In more developed proglottids, proliferating cells are abundant in the genital primordia and testes primordia. At least some of these cells must belong to the germ line, which is probably determined by epigenesis [42]. In late genital primordia, proliferating cells are restricted to the periphery.

### Comparison with previous studies in *M. corti*

The localization of cells in S and M phases determined in this work is very similar to the localization of "basophilic cells" described by Hess [28] in tetrathyridia. Subsequently, in an ultrastructural study, Hess [29] divided the pool of germinative cells in two subpopulations: "dark" germinative cells, localized close to the inner muscular layer and in which no mitoses were recorded, and "light" germinative cells, localized throughout the parenchyma, and in which mitoses were observed. Hess proposed that dark cells were the progeny of light cells, differentiating into parenchymal muscle cells. Although in our work the germinative cells were not characterized at the ultrastructural level, it is clear that the germinative cells localized close to the inner muscle layer proliferate actively.

In a previous BrdU incorporation study in tetrathyridia [31], labeled cells were found in the sub-tegumentary regions. Here, we demonstrate that this was probably due to the duration of the BrdU pulse (24 hours) since no cells are labeled in this region after 4 hours, while some (few) are seen after a 24 hour pulse. BrdU pulse and chase experiments further indicate that cell renewal in this region occurs by immigration of cells from the medullary parenchyma.

Espinoza *et al*. [32,33], based in autoradiographic analyses of tritiated thymidine incorporation in longitudinal sections, proposed that during segmentation, proliferating cells are localized and/or incorporated preferentially in the main nerve cords. Although their results with longitudinal sections are very similar to our own, the incorporation of different section planes, phalloidin staining and whole-mount detection of BrdU incorporation in this work shows that the distribution of proliferative cells is not restricted to the main nerve cords but is found throughout the periphery of the medullary parenchyma, close to the inner muscle layer. The distribution of proliferative cells in the scolex, next to the inner muscle layers, is congruent with this. Furthermore, during segmentation and formation of the genital primordia, proliferative cells accumulate in the dorsal and ventral regions of the medullary parenchyma, and not in the lateral regions, where the main nerve cords are located [37-39]. However, minor medial nerve cords are present in these regions, and there is an intimate relationship between the nerve cords and the muscle layer [37,38], so the ring of proliferative cells is actually close to both the nerve cords and the inner muscle layer.

Our results show no support for the apical massif [30] as a particularly proliferative region under our culture conditions. Both BrdU+ cells after a short (4 hour) pulse, and mitotic figures, are abundant throughout the scolex, including the region of the apical massif, but even in this region very few mitoses are found in close proximity to the sub-tegumental tissue, while most are close and inner to the dorso-ventral muscles that continue from the inner longitudinal muscle layer. It is possible that some of these mitotic cells belong to the apical massif, and that the apical massif has a more important role in cell proliferation during asexual reproduction of tetrathyridia, which was not studied in this work, but was studied in detail by Hess [30].

Results with BrdU pulse and chase experiments also suggest that during normal growth, and during development into segmented worms, cells in the sub-tegumental region (tegumental cells and sub-tegumental muscle cells) originate from germinative cells located throughout the body, as suggested by Smith and McKerr [31], and not from the apical massif. Experimental amputation of the scolex and apical massif followed by BrdU labeling further confirms this suggestion, since it demonstrates that proliferating cells from the medullary parenchyma of the body can be the source of cells in the sub-tegumental region. If the original source of these proliferating cells was the apical massif, then these cells would have to be able to remain undifferentiated and proliferating outside of the apical massif after two days of culture. However, no evidence was provided by Hess [30] for proliferation of the proposed migratory cells from the apical massif; only cells in the apical massif itself and germinative cells in the parenchyma were reported to incorporate tritiated thymidine.

### Comparison with other model cestodes

Although variation exists in the distribution of proliferative cells in larval forms [18,19], there appears to be a common pattern in the distribution of proliferative cells in the neck and proglottids of strobilizing cestodes. Proliferative cells are found mainly or exclusively in the external region of the medullary parenchyma, close to the inner muscle layer in *Diphyllobothrium dendriticum*, *Diphyllobothrium latum*, *Cylindrotaenia dispar*, *Hymenolepis diminuta *and *Taenia solium *[9,12,16,18,20]. In *Diphyllobothrium dendriticum *the distribution appears to be more disperse, with proliferative cells being present in the inner regions of the cortical parenchyma, although in the related *Diphyllobothrium latum *the distribution is more restricted [9]. We suggest that this is a conserved developmental characteristic of eucestodes. The renewal of the tegumental syncytium by mesenchymally originating stem cells has been proposed to be homologous to epidermal replacement in free-living flatworms, and seems to be unique for platyhelminthes [43] and the phylogenetically controversial Acoela [44]. On the other hand, proliferation within the scolex has been much less studied. In the case of *Hymenolepis diminuta*, no proliferative activity was detected in the scolex [18], in sharp contrast with our results in *M. corti*.

The distribution of proliferative cells next to the inner muscle layer, the development of the genital primordium from dorso-ventral thickenings of the ring of germinative cells (resulting in a genital primordium that traverses the medullary parenchyma), and the later appearance of the testes primordia laterally to the genital primordium are very similar between *M. corti *and *Cylindrotaenia dispar *(syn. *Baerietta dispar *[16,45]). These developmental similarities could be related to the fact that families Mesocestoididae and Nematotaeniidae have been proposed to be phylogenetically close, forming a monophyletic group at the base of the cyclophyllideans [46]. However, the phylogenetic position of Mesocestoididae is uncertain, and may in fact be basal to all other cyclophyllideans [47]. On the other hand, although the early genital primordium does not transverse all the medullary parenchyma in *D. dendriticum*, the posterior development of it is similar with *M. corti*, since in both cases the cells of the inner region adopt a compact disposition and cease to proliferate [11]. Cell proliferation in genital primordia has not been reported, to the best of our knowledge, in other eucestode species, but this could be another conserved developmental characteristic of eucestodes.

## Conclusions

In this work, we have thoroughly characterized proliferative cells during the *in vitro *development of *M. corti *from tetrathyridium to young segmented worm by two different methods with congruent results, which are similar to those described in other model cestodes. Throughout development, cell proliferation in the body only occurs in the medullary parenchyma; cell renewal in the cortical parenchyma and sub-tegument occurs by migration and integration of cells from the medullary parenchyma. *In situ *proliferation contributes to the formation and growth of segmental and genital primordia, and diminishes during the differentiation of the latter. We conclude that the localization and characteristics of proliferative cells are evolutionary conserved between *Mesocestoides *and other model cestodes, and are compatible with a stem cell nature of these cells.

## Methods

### Parasite culture

Mice infected with *M. corti *tetrathyridia were kindly donated by Laura Dominguez and Jenny Saldaña (Facultad de Química, Uruguay). Parasite removal and *in vitro *culture was performed as described by Britos *et al*. [48]. Briefly, approximately 500 μl of tetrathyridia were cultured in 15 ml of modified RPMI 1640 with 10% bovine fetal serum. (RPMI 1640 media, HEPES modified (Sigma-Aldrich) with 4.3 g/l glucose, 4.8 g/l yeast extract and 50 μg/ml gentamycin added) at 37°C under a 5% CO2 atmosphere. Two thirds of the medium was replaced every 48 to 72 hours. For induction of strobilization, tetrathyridia were cultured for 48 hours and then sodium taurocholate (ST; Sigma-Aldrich) was added to a final concentration of 1 mg/ml, maintaining this concentration in successive media changes. Under these conditions, strobilar development is apparent 5 to 7 days after the induction with ST.

### Whole-mount staining with TO-PRO-3

Worms were fixed overnight at 4°C with 4% paraformaldehyde prepared in PBS (PFA-PBS). After exhaustive washes in PBS, they were stained with TO-PRO-3 (Invitrogen; 0.2 μM in PBS) for one hour, washed five times with PBS for ten minutes each and mounted in ProLong Gold Antifade Reagent (Invitrogen).

### Incorporation of 5-Bromo-2'-Deoxyuridine (BrdU)

Samples of cultures of *M. corti *were cultured in RPMI-1640 modified media without yeast extract and with 10% bovine fetal serum, containing 5 mM BrdU (Sigma-Aldrich), for 4 hours or for 24 hours. Some individuals were fixed immediately after the BrdU pulse, while the rest were washed exhaustively with RPMI-1640 modified medium without BrdU and cultured in the same medium until 72 hours had passed since the beginning of BrdU incubation. Experiments were performed with un-induced tetrathyridia, tetrathyridia induced for 48 hours with ST, and segmented worms after at least 6 days of induction with ST.

For acephalic fragments, tetrathyridia were amputated with a sterile blade under a dissecting microscope, washed extensively with sterile PBS supplemented with 50 μg/ml gentamycin, 100 units/ml penicillin, 100 μg/ml streptomycin, and cultured for two days in BrdU-free media supplemented with the same antibiotics. Then, they were incubated for 3 days in RPMI-1640 modified media without yeast extract and with 10% bovine fetal serum, containing 250 μM BrdU.

### Immunohistofluorescent and immunohistochemical detection of BrdU

Detection was performed both in whole-mounts and in serial sections. In the case of whole-mounts, the tegument was removed by incubating the live specimens in distilled water for 5 hours as described by Gustafsson [49] before fixing with PFA-PBS. Similarly to what was described by Gustafsson [49] for other cestode species, worms elongated considerably in addition to loosing the tegument. Staining with TO-PRO-3 confirmed that only the tegument was lost while the nuclei in the sub-tegumental region remained and that the genital primordia and testis primordia were well conserved and morphologically recognizable [see Additional File 5]. Specimens were then fixed either overnight at 4°C or for 4 hours at room temperature with PFA-PBS, dehydrated progressively to 100% ethanol and stored at -20°C until further use. After rehydration, the following detection protocol was performed: specimens were treated with protease K (20 μg/ml) in PBS for 20 minutes at room temperature, washed in PBS, and treated with HCl 2 N in PBS for 30 to 60 minutes at room temperature. After several washes in PBS, specimens were incubated with PBS plus 0.1% Triton X-100 (two washes of 15 minutes each), washed once in PBS for 1 minute, blocked with 1% bovine serum albumin (BSA) and 5% sheep serum (Sigma-Aldrich) in PBS plus 0.05% Tween-20 for one hour, and incubated with anti-BrdU monoclonal antibody (Sigma-Aldrich, 1:100 dilution) in PBS with 1% BSA and 0.05% Tween-20 for two hours at room temperature. Samples were then washed six times, 20 minutes each, with PBS plus 0.05% Tween-20 (PBS-Tw). For immunohistofluorescent detection, samples were incubated with goat anti-mouse antibody conjugated to Cy5 (Chemicon), diluted 1:800 in PBS with 1% BSA and 0.05% Tween-20, for 1 hour at 37°C. Specimens were finally washed six times, 20 minutes each, with PBS-Tw, and mounted with ProLong Gold AntiFade Reagent (Invitrogen). Negative controls of specimens not incubated in the presence of BrdU did not show any signal. For immunohistochemical detection, samples were incubated with polyclonal goat anti-mouse antibody conjugated to horse radish peroxidase (Dako) diluted 1:100 in PBS with 1% BSA, and detected with 3-Amino-9-ethylcarbazole (AEC, Sigma-Aldrich).

For serial sections, specimens were embedded in Paraplast (Oxford Labware) as described by the manufacturer and cut in 10 μm thick sections. The detection protocol was similar to the protocol for whole-mount specimens, except that protease K concentration was reduced to 1 μg/μl and washes were reduced in length by half.

TO-PRO-3 stained worms and immunohistofluorescent detection of BrdU were analyzed with an Olympus FV300 confocal microscope.

### Identification of mitoses in paraffin sections of specimens treated with colchicine

*M. corti *specimens that had been cultured for 6 days were incubated in RPMI-1640 modified media with 0.05% colchicine at 37°C for 6 hours. Fixation and sectioning were performed as described above into sections of 6 or 10 μm. Sections were stained with 4',6-diamidino-2-phenylindole (DAPI) dilactate (Sigma-Aldrich; 1 μg/ml in PBS) with or without ethidium bromide (Sigma-Aldrich; 1 μg/ml in PBS) for 15 minutes, washed 4 times for 5 minutes each with PBS, and mounted in ProLong Gold Antifade Reagent (Invitrogen). The slides were analyzed with a fluorescence microscope (Olympus). Over 600 mitoses were identified from 35 longitudinal sections from six different specimens.

### Identification of mitoses in cryosections and phalloidin staining

*M. corti *specimens were fixed for 90 minutes in PFA-PBS at room temperature, washed extensively with PBS, and incubated in 30% saccharose at 4°C for 48 hours. Specimens were embedded in Tissue-*Tek *OCT compound, frozen with liquid nitrogen, and sections 10 μm thick were obtained with a cryostat, and mounted on SilanePrep slides (Sigma-Aldrich). Staining with Alexa-546 conjugated phalloidin (Invitrogen) was performed as instructed by the manufacturer. Co-staining with DAPI was performed as described above.

## Competing interests

The authors declare that they have no competing interests.

## Authors' contributions

UK carried out or participated in all experiments. MFD performed parasite cultures, and participated in BrdU labeling experiments and histology. UK and EC designed the study and drafted the manuscript. MM and AK participated in discussions and experimental design, and contributed with essential reactives. All authors read and approved the final manuscript.

## Supplementary Material

Additional file 1 **Supplementary figure 1: Whole-mount BrdU detection after a 24 hour pulse in un-induced tetrathyridia**. A. Anterior region of tetrathyridium. B. Medium and posterior region of tetrathyridium. C. Scolex showing BrdU+ cells within suckers (broken circles). Bars represent 100 μm in A, and 200 μm in B and C. Click here for file

Additional file 2 **Supplementary figure 2: Histological detection of mitoses in colchicine treated specimens stained with ethidium bromide (red) and/or DAPI (blue)**. A, B, C. Sagittal sections. Arrows in all figures indicate mitotic cells. *cp*, cortical parenchyma; *g*, germinative cells; *gp*, genital primordium; *iml*, inner muscle layer; *m*, dorso-ventral muscle cell; *mp*, medullary parenchyma; *st*, sub-tegument; *t*, tegument. Bars represent 10 μm. Click here for file

Additional file 3 **Supplementary figure 3: Histological detection of mitoses within the suckers**. Specimens were stained with ethidium bromide (red) and DAPI (blue). Arrows indicate mitotic cells. *s*, sucker. Insets show close-ups of mitotic cells. Bars represent 10 μm. Click here for file

Additional file 4 **Supplementary figure 4: Histological detection of mitoses in the anterior-most regions of the scolex**. Specimens were stained with ethidium bromide (red) and DAPI (blue). A, B. Sagittal sections. Anterior is to the bottom. Insets show general views of the scolex stained with DAPI. Arrows indicate mitotic cells. *s*, sucker; *exc*, excretory ducts. Bars represent approximately 10 μm. Click here for file

Additional file 5 **Supplementary figure 5: Morphology of *M. corti *after tegument removal**. Specimens were stained with TO-PRO-3. A. Close-up of the border of the body wall; the broken line indicates the position of the inner muscle layer. B. Segment showing the genital primordium (arrow) and testes primordia (asterisks). Bars represent 50 μm. Click here for file
