# Supplementary figures and images for: Stem cell proliferation during in vitro development of the model cestode Mesocestoides corti from larva to adult worm
Source: Front Zool. 2010 Jul 13;7:22. doi: 10.1186/1742-9994-7-22 (PMC2917415; doi:10.1186/1742-9994-7-22)

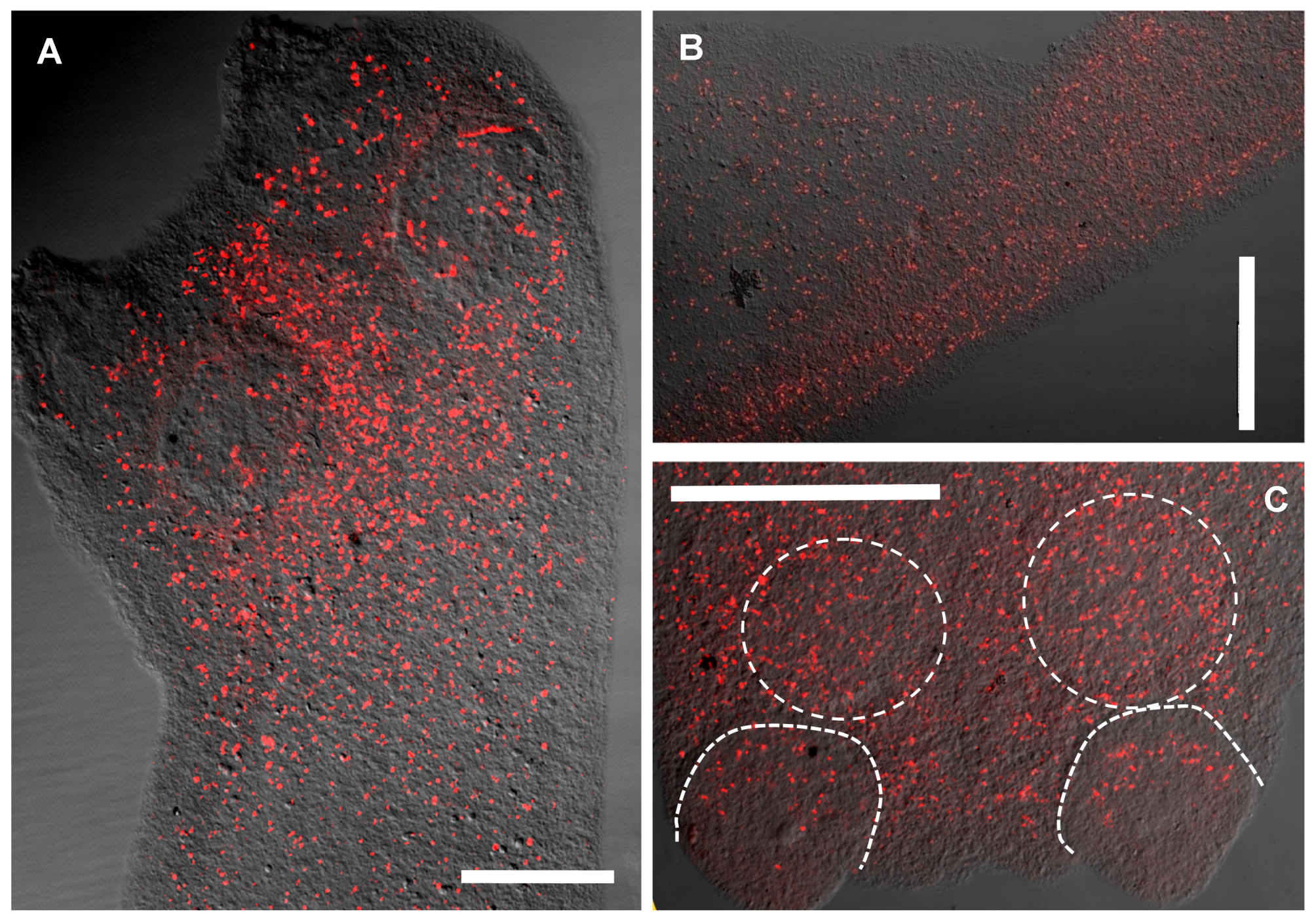

Supplement: Additional file 1 — Supplementary figure 1: Whole-mount BrdU detection after a 24 hour pulse in un-induced tetrathyridia. A. Anterior region of tetrathyridium. B. Medium and posterior region of tetrathyridium. C. Scolex showing BrdU+ cells within suckers (broken circles). Bars represent 100 μm in A, and 200 μm in B and C. [file 1742-9994-7-22-S1.tiff]

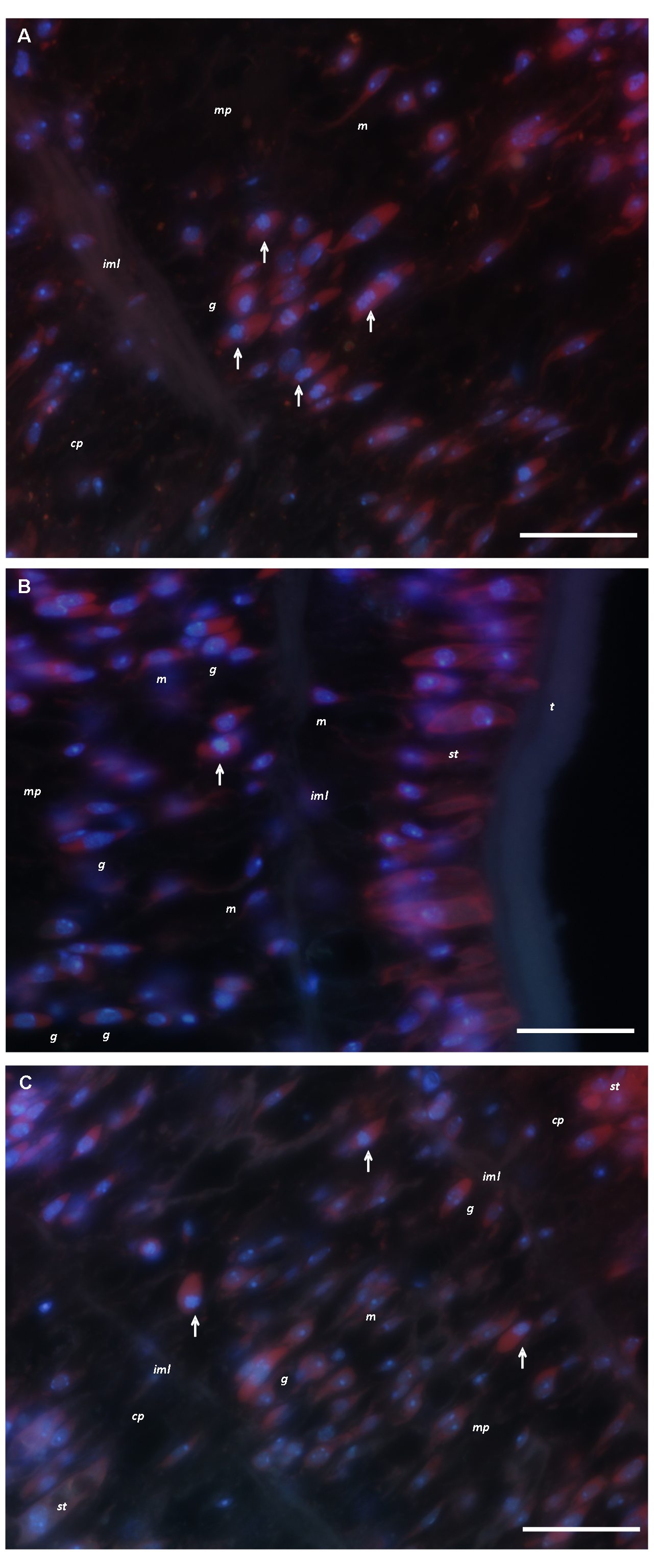

Supplement: Additional file 2 — Supplementary figure 2: Histological detection of mitoses in colchicine treated specimens stained with ethidium bromide (red) and/or DAPI (blue). A, B, C. Sagittal sections. Arrows in all figures indicate mitotic cells. cp, cortical parenchyma; g, germinative cells; gp, genital primordium; iml, inner muscle layer; m, dorso-ventral muscle cell; mp, medullary parenchyma; st, sub-tegument; t, tegument. Bars represent 10 μm. [file 1742-9994-7-22-S2.tiff]

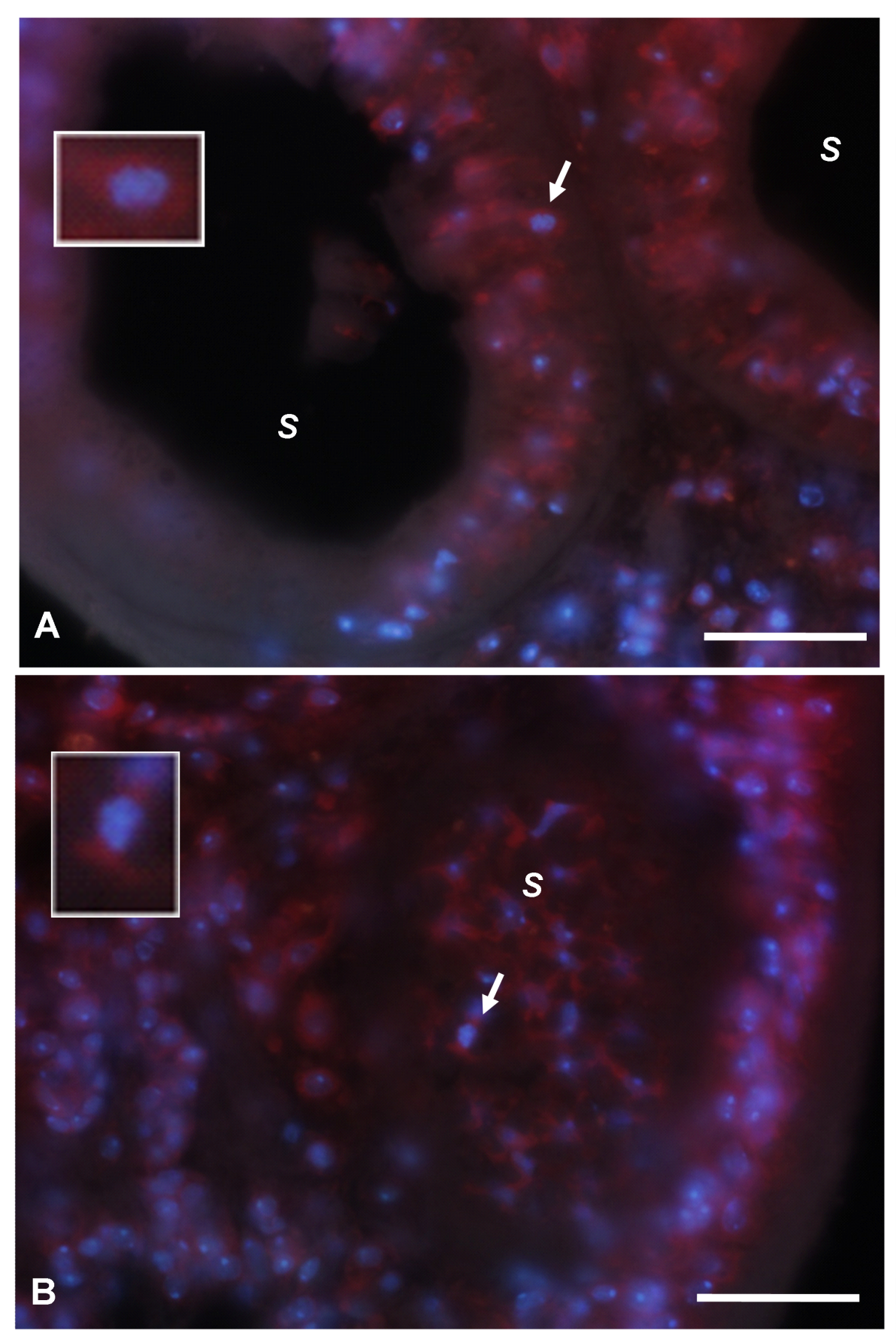

Supplement: Additional file 3 — Supplementary figure 3: Histological detection of mitoses within the suckers. Specimens were stained with ethidium bromide (red) and DAPI (blue). Arrows indicate mitotic cells. s, sucker. Insets show close-ups of mitotic cells. Bars represent 10 μm. [file 1742-9994-7-22-S3.tiff]

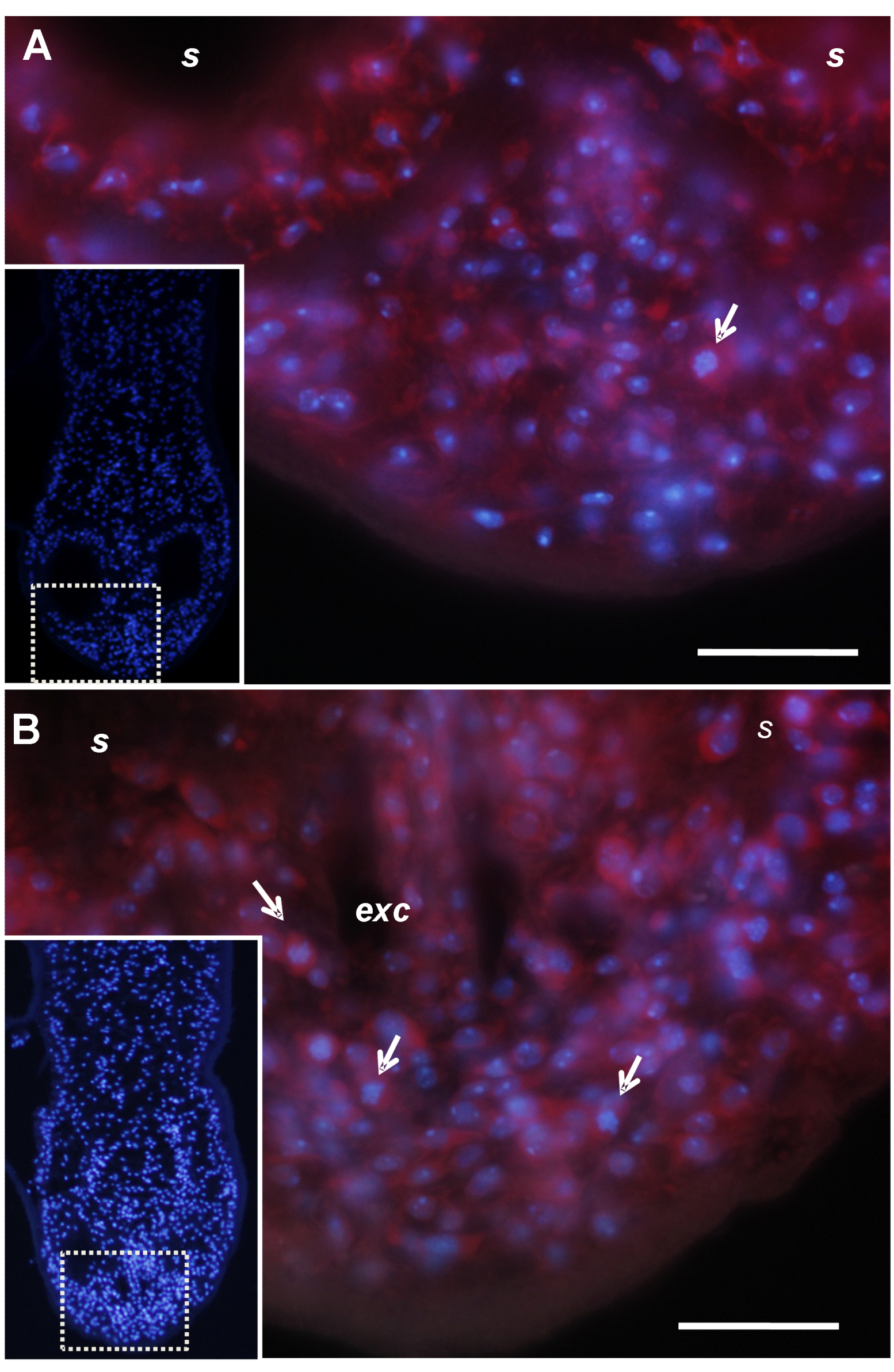

Supplement: Additional file 4 — Supplementary figure 4: Histological detection of mitoses in the anterior-most regions of the scolex. Specimens were stained with ethidium bromide (red) and DAPI (blue). A, B. Sagittal sections. Anterior is to the bottom. Insets show general views of the scolex stained with DAPI. Arrows indicate mitotic cells. s, sucker; exc, excretory ducts. Bars represent approximately 10 μm. [file 1742-9994-7-22-S4.tiff]

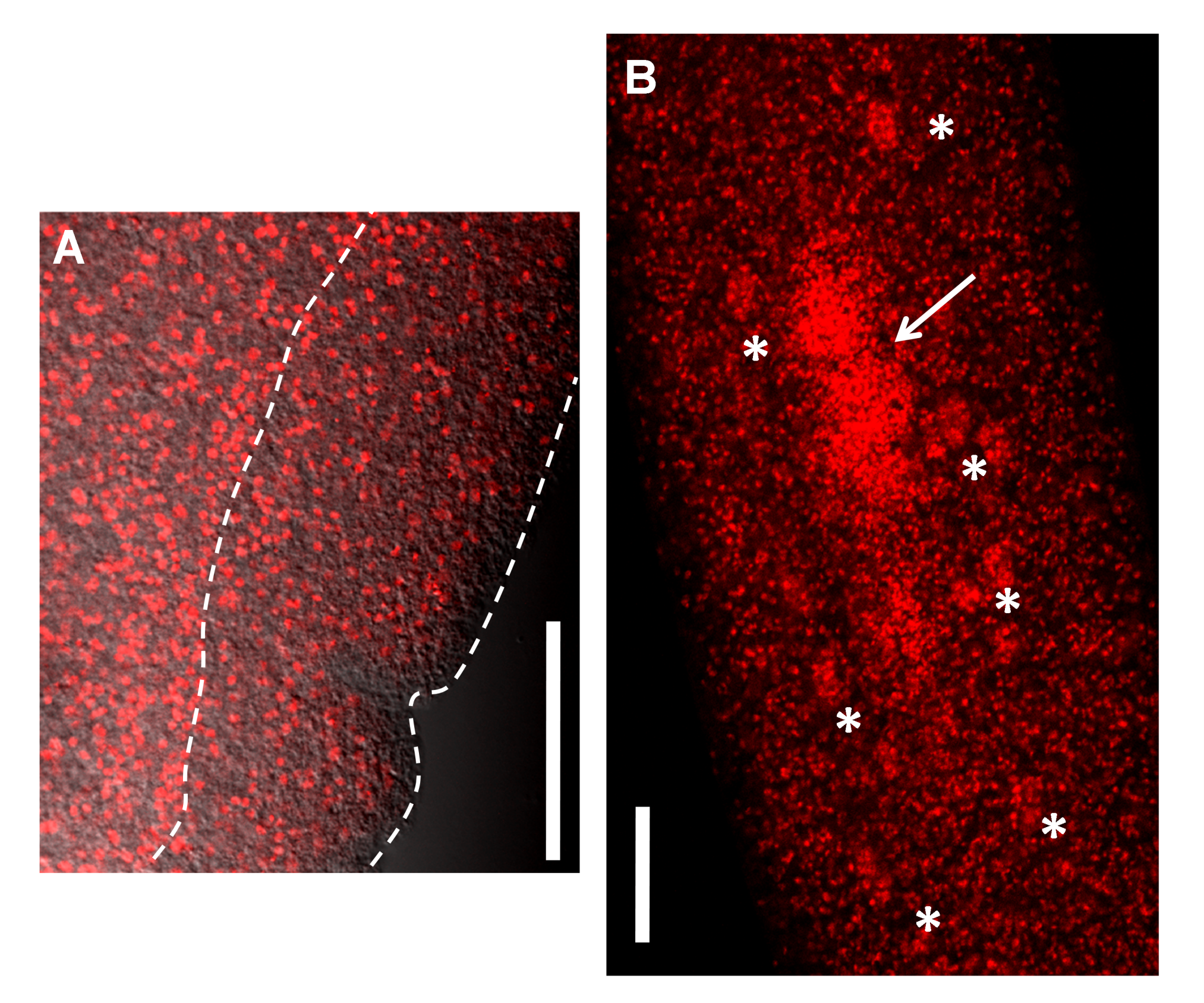

Supplement: Additional file 5 — Supplementary figure 5: Morphology of M. corti after tegument removal. Specimens were stained with TO-PRO-3. A. Close-up of the border of the body wall; the broken line indicates the position of the inner muscle layer. B. Segment showing the genital primordium (arrow) and testes primordia (asterisks). Bars represent 50 μm. [file 1742-9994-7-22-S5.tiff]
